# Pre-hospital delay > 7 days independently predicts impaired wound healing in diabetic foot ulcer patients: a 3-year retrospective cohort study

**DOI:** 10.3389/fendo.2026.1860867

**Published:** 2026-07-14

**Authors:** Yue Zhang, Xinxiu Huang, Jing Li, Shaogang Ma, Bing Zhang

**Affiliations:** 1 Department of Clinical Laboratory, The People’s Hospital of Laibin, Laibin, Guangxi Autonomous Region, China; 2 Department of Endocrinology and Metabolism, The People’s Hospital of Laibin, Laibin, Guangxi Autonomous Region, China; 3 Department of Endocrinology and Metabolism, Suqian First Hospital, Suqian, Jiangsu, China; 4 Department of Hand and Foot Surgery, The People’s Hospital of Laibin, Laibin, Guangxi Autonomous Region, China

**Keywords:** assessment, clinical characteristics, diabetic foot ulcers, outcomes, timely medical attention

## Abstract

**Background:**

Diabetic foot ulcers (DFUs) are a severe, costly complication of diabetes and a major public health challenge. Early multidisciplinary intervention is critical. This study investigated how pre-hospital practices affect clinical characteristics, metabolic profiles, and outcomes in DFU patients.

**Methods:**

A retrospective cohort study included DFU patients treated at a tertiary center (2021-2024). Patients were stratified into the timely medical care group (TMCG, ≤ 7 days) and the delayed medical care group (DMCG, 7–30 days) based on initial consultation timing. Comparative analysis evaluated metabolic parameters and lesion severity. Kaplan-Meier analysis was performed to assess healing outcomes, and multivariable Cox regression was used to identify predictors of DFU healing.

**Results:**

A total of 116 patients were enrolled in this study. A high proportion of patients in both the TMCG (n = 49) and DMCG (n = 67) had used Chinese herbal medicine or topical Western medications prior to admission. Compared to patients in the TMCG, those in the DMCG were associated with more severe Wagner grades (≥ 3: 38.8% vs. 18.4%, *p* = 0.02), hypoalbuminemia (< 35 g/L: 62.7% vs. 40.8%, *p* = 0.02), and higher rates of surgical intervention (14.9% vs. 4.1%, *p* = 0.02). Kaplan-Meier analysis revealed that the DMCG had a significantly higher risk of non-healing than the TMCG (*p* = 0.002). Multivariate Cox regression analysis identified delayed medical care (7–30 days) (HR = 1.62, 95% CI: 1.02–2.56, *p* = 0.04), and a higher Wagner grade (HR = 1.62, 95% CI: 1.19–2.22, *p* = 0.003) as independent predictors of impaired DFU healing.

**Conclusions:**

Inappropriate pre-hospital practices commonly delay definitive care and worsen outcomes. Early medical intervention (≤ 1 week) is positively associated with therapeutic outcomes and reduces the risk of complications.

## Introduction

Diabetic foot ulcers (DFUs) are a severe complication of diabetes mellitus, often resulting from a combination of risk factors such as prolonged pressure, thermal injuries, neuropathy, and peripheral arterial disease. Among the 537 million people worldwide with type 2 diabetes, 19% to 34% are expected to develop a DFU in their lifetime (1, 2), and this number is rising with increased longevity and medical complexity in the diabetic population (3). DFUs, a complex and preventable condition, are the most disabling sequelae, significantly contributing to patient morbidity, lower-limb amputations, and high healthcare costs (1).

Despite the recognized importance of early intervention, treatment-seeking delay remains common among DFU patients. Pre-hospital delay is defined as the period from initial foot lesion detection to formal specialist consultation. Lingyan et al. confirmed that delayed referral is closely associated with aggravated ulcer severity and poor limb salvage outcomes (4). According to Abbas et al., a median pre-hospital delay of seven days was mainly caused by financial constraints and inadequate disease awareness (5).

However, most previous studies treated delay as a continuous variable or applied ambiguous grouping without a clinically feasible critical cutoff. Three recent domestic investigations further highlight unresolved research gaps. Zhu et al. adopted a 7-day boundary to classify patients into <7 days, 7–14 days and >14 days, and demonstrated that prolonged delay raises surgical risk and medical costs, yet failed to address prevalent self-medication practices in China (6). Ge et al. observed an average pre-hospital delay of five months in Chinese DFU cohorts and linked delayed consultation to poor health literacy and insufficient primary care resources, without quantitative analysis of delay against wound healing indexes (7). Xu et al. merely explored psychosocial risk factors for delayed medical help (8).

Although numerous studies have focused on in-hospital DFUs management, few studies quantify the combined influence of pre-hospital delay and informal self-medication on patients’ metabolism, admission wound severity and definitive healing endpoints, and scarcely validate whether delay over 7 days independently predicts poor prognosis with the standardized 7-day threshold. Accordingly, we conducted this retrospective cohort study to clarify whether pre-hospital delay >7 days independently worsens metabolic parameters and Wagner grade and serves as an independent risk factor for unsatisfactory DFU healing.

## Methods

### Study design and ethical approval

This retrospective cohort study evaluated consecutive patients with DFUs admitted to The People’s Hospital of Laibin, a tertiary referral center in Guangxi Autonomous Region, China, between January 2021 and December 2024. The diabetic foot center at this institution provided multidisciplinary care integrating endocrinology, podiatric surgery, and nutritional support. All diagnoses were coded according to the International Classification of Diseases, 10th Revision (ICD-10), and confirmed by specialist assessment upon admission. Given the retrospective study design, data were collected from routine clinical practice. Because individual informed consent was not obtained, the ethical committee waived the requirement for informed consent, and all patient data were anonymized.

The study was approved by the Ethics Committee of The People’s Hospital of Laibin (No. 2023-21) (September 12, 2023) and registered with No. ChiCTR2300077025 (October 26, 2023). It was conducted in accordance with the principles outlined in the Declaration of Helsinki.

### Inclusion and exclusion criteria

The first diagnosis must comply with the disease codes for DF/DFUS (ICD-10: E11.704/E10.702), with screening conducted according to specific clinical circumstances. Consecutive patients meeting the inclusion criteria were enrolled to minimize selection bias. The inclusion criteria were as follows: (1) diagnosis of DFUs established according to the 2019 International Working Group on the Diabetic Foot (IWGDF) guidelines (9); (2) age ≥ 18 years; (3) newly diagnosed with diabetic foot ulcers or first-ever hospitalization for DFUs at our center; and (4) documented onset date of foot ulceration and first medical consultation date, allowing calculation of pre-hospital delay. The exclusion criteria were as follows: (1) lack of clinical data at admission; (2) patients with refractory DFUs despite recurrent hospital admissions. Given the single-center, retrospective nature of the study, the sample comprises consecutive eligible patients within the study period rather than a sample representative of the general population.

### Data sources and clinical assessments

Data were extracted from the electronic medical record system using a standardized abstraction form. Two researchers completed independent double data entry to ensure data quality. Variables are defined as follows: (1) timely Medical Care Group (TMCG): initial consultation at our center ≤7 days from self-reported ulcer onset; delayed Medical Care Group (DMCG): initial consultation 7–30 days from onset; (2) Wagner grade: assessed by the attending podiatric surgeon (B.Z.) at admission based on wound depth, infection status, and presence of gangrene, using the standard Wagner classification system (9); (3) healing: complete epithelialization without drainage or need for further surgical intervention, confirmed at 1-year follow-up visit by the podiatric surgery team; (4) hypoalbuminemia: serum albumin <35 g/L, based on the institutional reference range; (5) hyperlipidemia: documented history in the medical record or admission lipid profile meeting the National Cholesterol Education Program Adult Treatment Panel III criteria; (6) surgical intervention: any procedure requiring operating theater admission, including major amputation, revascularization, or extensive debridement under general anesthesia.

### Statistical analysis

All statistical analyses were performed using SPSS 26.0. A retrospective sample size check was conducted to verify adequate statistical power. Among the clinical and laboratory parameters, normally distributed data were summarized with mean ± standard deviation and analyzed using the *t*-test. Non-normal data were summarized with median (interquartile range) and analyzed using the Mann-Whitney U test. Categorical variables were expressed as frequencies and percentages (%) and compared with the chi-squared test or Fisher’s exact test, as appropriate. Kaplan-Meier analysis with the log-rank test was selected to compare time to non-healing between TMCG and DMCG. Patients who were lost to follow-up or transferred to other institutions before achieving healing or unhealed were censored at their last documented assessment. Subsequently, patient outcomes were classified into healing and non-healing groups, and clinical characteristics and laboratory indicators were compared between them. Multivariable Cox proportional hazards regression was employed to estimate hazard ratios for non-healing while adjusting for potential confounders. A *p*-value < 0.05 was considered statistically significant.

## Results

### Pre-hospital management ranking

The ranking of pre-hospital management approaches, from most to least frequent, was as follows: no treatment, Chinese herbal medicine, topical Western medication, antibiotic infusion and debridement at community clinics, with other methods remaining unspecified. Both the TMCG and DMCG showed a high proportion of patients choosing topical medications. Furthermore, in the DMCG, there was a significantly higher proportion of patients using topical traditional Chinese medicine, topical Western medicine, and other unspecified methods compared to the TMCG (86.6% vs. 61.2%, *p* = 0.002), resulting in a higher rate of untreated cases (**Table 1**).

**Table 1:** A Summary of various treatments undergone prior to admission to the DFU center.

Variables	TMCG	DMCG	P-value
	(n = 49)	(n = 67)	
No treatment, n (%)	19 (38.8)	9 (13.4)	0.002
Unprofessional treatment, n (%)	30 (61.2)	58 (86.6)	
Topical Chinese herbal medicine, (n)	8	18	
Topical Western medication, (n)	14	17	
Antibiotic administration via infusion at community clinics, (n)	1	2	
Bandaging at non-diabetic foot centers, (n)	1	5	
Unspecified treatment, (n)	6	16	

### Demographic and metabolic characteristics

A total of 116 patients with DFUs were included in the study, with 49 patients in the TMCG and 67 patients in the DMCG. No significant differences were observed between the TMCG and DMCG groups in terms of gender, age, duration of diabetes, prevalence of hypertension, thyroid function, insurance type, or electrolyte disorders. Compared with the TMCG, the DMCG showed a longer duration from ulcer onset to presentation (20.71 ± 8.22 vs. 4.40 ± 2.20, *p* < 0.001) (this was solely due to the grouping criteria themselves and carries no additional clinical interpretive value), a lower proportion of patients with hyperlipidemia (40.4% vs. 49.0%, *p* = 0.01), lower hemoglobin concentration (104.58 ± 21.98 vs. 112.81 ± 19.57, *p* = 0.04), higher platelet levels (310.22 ± 89.50 vs. 273.35 ± 75.20, *p* = 0.02), and a higher prevalence of hypoalbuminemia (62.7% vs. 40.8%, *p* = 0.02) (**Table 2**).

**Table 2:** Comparison of general clinical data and biochemical examination indicators between TMCG and DMCG groups.

Variables	TMCG (n = 49)	DMCG (n = 67)	*P*-value
Gender (M/F)	29/20	43/24	0.69
Age (years)	64.82 ± 9.64	65.99 ± 11.17	0.55
SBP (mmHg)	136 ± 24	135 ± 25	0.85
DBP (mmHg)	77 ± 14	80 ± 13	0.18
Duration of diabetes (years)	9.07 ± 6.66	7.88 ± 5.81	0.32
Duration of DFUs (days)	4.40 ± 2.20	20.71 ± 8.22	<0.001
Hyperlipidemia, n (%)	24 (49.0)	52 (40.4)	0.01
Hypertension, n (%)	34 (69.4)	39 (58.2)	0.21
Chronic kidney disease, n (%)	32 (65.3)	41 (61.2)	0.65
Plasma glucose on admission (mmol/L)	17.21 ± 7.65	17.93 ± 8.82	0.65
HbA1c (%)	10.30 ± 3.12	10.97 ± 3.35	0.32
Health insurance type (employee/resident)	11/37	10/56	0.29
Urine microalbumin-to-creatinine ratio	11.50 (63.65)	17.04 (91.92)	0.30
C-reactive protein (mg/L)	43.11 (82.49)	6.85 (141.60)	0.40
FT3 (pmol/L)	3.40 ± 1.25	3.15 ± 0.95	0.29
FT4 (pmol/L)	15.43 ± 2.81	15.82 ± 2.92	0.52
TSH (mIU/L)	2.61 ± 1.94	2.34 ± 1.82	0.50
White blood cell count (×10^9^)	10.62 (12.35)	8.02 (3.65)	0.33
neutrophil count (%)	71.05 ± 13.15	69.47 ± 13.78	0.54
Erythrocyte count (×10^12^)	4.02 ± 0.65	3.77 ± 0.90	0.11
Hemoglobin (g/L)	112.81 ± 19.57	104.58 ± 21.98	0.04
Platelet count(×10^9^)	273.35 ± 75.20	310.22 ± 89.50	0.02
K**^+^** (mmol/L)	4.17 ± 0.72	4.05 ± 0.59	0.34
Hypokalemia, n (%)	6 (12.2)	10 (14.9)	0.69
Na**^+^** (mmol/L)	136.53 ± 5.44	136.68 ± 5.56	0.88
Hyponatremia, n (%)	16 (32.7)	17 (25.6)	0.37
Total Ca^2^**^+^** (mmol/L)	2.22 ± 0.16	2.21 ± 0.16	0.71
Cl^-^ (mmol/L)	100.95 ± 6.51	100.97 ± 6.62	0.98
Mg^2^**^+^ (**mmol/L)	0.84 ± 0.18	0.84 ± 0.11	0.98
P (mmol/L)	1.11 ± 0.30	1.10 ± 0.32	0.86
Serum uric acid (μmol/L)	94.50 (148.72)	92.20 (119.00)	0.83
Serum creatinine (μmol/L)	94.50 (74.30)	86.00 (87.10)	0.57
Serum Albumin (g/L)	35.02 ± 5.66	33.37 ± 6.06	0.14
Total Serum Protein (g/L)	66.01 ± 7.50	65.56 ± 9.06	0.78
Hypoalbuminemia, n (%)	20 (40.8)	42 (62.7)	0.02

Data are presented as numbers (percentages), mean ± standard deviation, or median values (interquartile range)P values represent results of t-tests or Mann–Whitney U test for continuous variables or the χ^2^ or Fisher test for categorical variables. SBP, systolic pressure; DBP, diastolic pressure; HbA1c, glycated Hemoglobin; FT3, free triiodothyronine; FT4, free thyroxine; TSH, thyroid-stimulating hormone; K^+^, serum potassium; Na^+^, serum sodium; Total Ca^2+^, serum total calcium; Cl^-^, serum chloride; Mg^2+^, serum magnesium; P, serum phosphorus.

Both groups exhibited several risk factors associated with diabetic foot complications, including an average age of nearly 65 years, a higher prevalence of hypertension, hyperlipidemia, and chronic kidney disease, a diabetes duration of nearly 10 years, and overall poor glycemic control. At admission, patients in both groups presented with elevated blood glucose levels and high glycated hemoglobin (HbA1c) levels, averaging around 10%. Additionally, signs of malnutrition were observed in both groups, characterized by mild anemia, low serum sodium, magnesium, and albumin levels (**Table 2**).

### Characteristics and managements of DFUs

**Figure 1:**
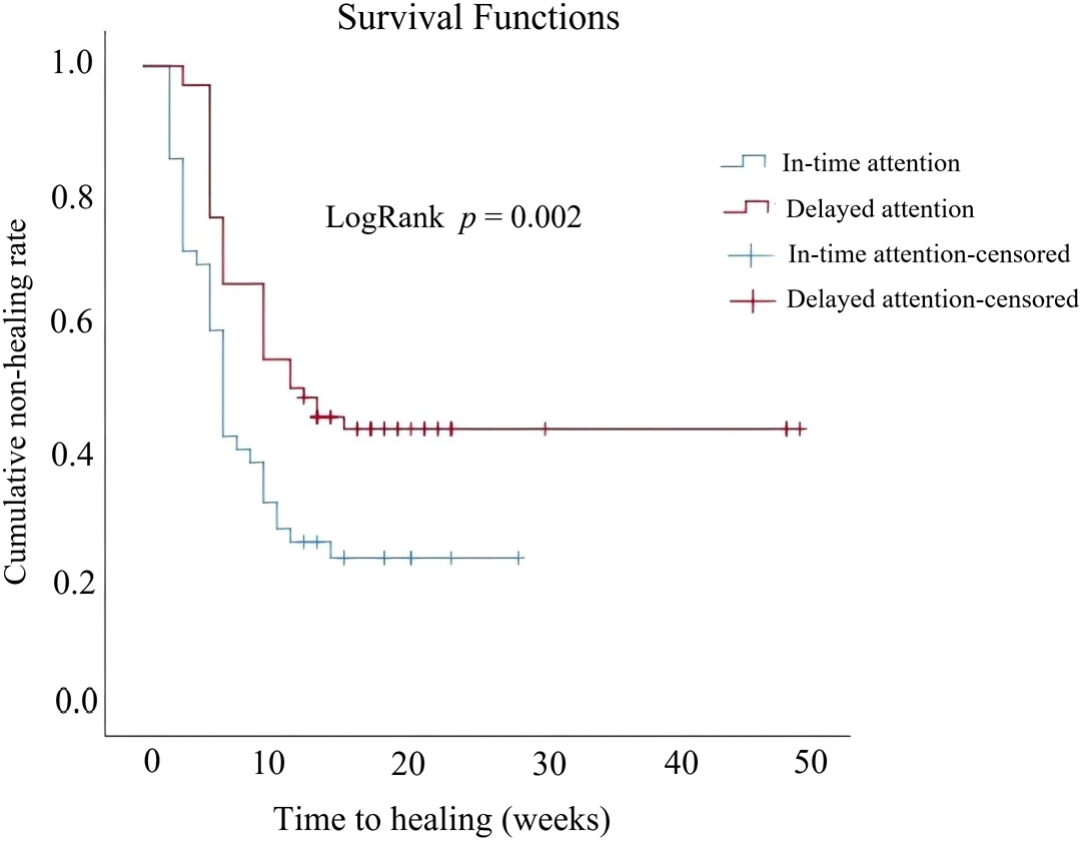
Kaplan-Meier curves comparing non-healing rates between delayed and timely admission groups.

The DMCG had significantly more patients with higher Wagner grades (3–5) compared to the TMCG (*p* = 0.02). The ulcer area was larger in the DMCG, but the difference did not reach statistical significance between the two groups. Surgical debridement and amputation rates were significantly higher in the DMCG (*p* = 0.02) (**Table 3**). Between the non-healing group and the healing group, there was a significant difference in diastolic pressure, duration of DFUs, HbA1c, C-reactive protein, free triiodothyronine (FT3), white blood cell count, hemoglobin, platelet count, Wagner grades, serum albumin (all *p* < 0.05) (**Table 4**). Survival analysis demonstrated a statistically significant higher cumulative non-healing rate in the DMCG (*p* = 0.002) (**Figure 1**).

**Table 3:** Comparison of foot lesion characteristics and 1-year outcomes between TMCG and DMCG groups.

Variables	TMCG (n = 49)	DMCG (n = 67)	*P*-value
Wagner grades			0.02
1–2 grades, n (%)	40 (81.6)	41 (61.2)	
3–5 grades, n (%)	9 (18.4)	26 (38.8)	
Ulcer area (cm^2^)	2.75 (10.25)	4.00 (14.00)	0.58
Sinus tract, n (%)	6 (12.2)	9 (13.4)	0.80
Negative pressure aspiration, n (%)	7 (14.3)	15 (22.4)	0.34
Surgical debridement, n (%)	12 (24.5)	22 (32.8)	0.41
Transfer to surgery, n (%)	2 (4.1)	10 (14.9)	0.02

The χ^2^ or Fisher test for categorical variables.

**Table 4:** Comparison of clinical characteristics and laboratory indicators between the unhealed and healed groups.

Variables	Non-healing group (n =40)	Healing group (n =76)	*p*-value
Gender (M/F)	22/18	49/27	0.37
Age (years)	63.38 ± 9.40	66.61 ± 11.03	0.11
SBP (mmHg)	130.15 ± 21.19	138.97 ± 25.53	0.06
DBP (mmHg)	75.72 ± 11.35	81.07 ± 14.23	0.04
Duration of diabetes (years)	8.45 ± 6.92	8.47 ± 5.87	0.98
Duration of DFUs (days)	19.08 ± 11.68	12.77 ± 10.54	0.004
Hyperlipidemia, n (%)	5 (12.5)	23 (30.3)	0.06
Hypertension, n (%)	24 (60.0)	49 (64.5)	0.71
Chronic kidney disease, n (%)	22 (55.0)	50 (65.8)	0.23
Plasma glucose on admission (mmol/L)	19.22 ± 9.30	16.75 ± 7.65	0.15
HbA1c (%)	12.22 ± 3.62	10.04 ± 2.84	0.003
Health insurance type (employee/resident)	3/35	18/60	0.06
C-reactive protein (mg/L)	75.20(153.86)	8.5(38.40)	<0.001
FT3 (pmol/L)	2.89 ± 1.11	3.47 ± 1.01	0.01
FT4 (pmol/L)	15.14 ± 2.97	15.97 ± 2.80	0.21
TSH (mIU/L)	1.92 ± 1.73	2.72 ± 1.90	0.06
White blood cell count (×10^9^)	12.95(10.81)	7.72(3.23)	0.004
neutrophil count (%)	75.20 ± 14.83	67.18 ± 11.90	0.005
Erythrocyte count (×10^12^)	3.78 ± 0.78	3.93 ± 0.82	0.36
Hemoglobin (g/L)	101.85 ± 20.17	111.72 ± 21.18	0.01
Platelet count(×10^9^)	321.10 ± 91.47	280.91 ± 79.73	0.01
Serum uric acid (μmol/L)	176.10(3022.75)	99.00(158.32)	0.19
Wagner grades			<0.001
1–2 grades, n (%)	16 (40.0)	64 (84.2)	
3–5 grades, n (%)	24 (60.0)	12 (15.8)	
Serum creatinine (μmol/L)	115.85(109.25)	85.00(70.60)	0.30
Serum Albumin (g/L)	32.14± 6.54	35.09 ± 5.33	0.01
Total Serum Protein (g/L)	66.64 ± 8.82	65.26 ± 8.21	0.41

Data are presented as numbers (percentages), mean ± standard deviation, or median values (interquartile range). P values represent results of t-tests or Mann–Whitney U test for continuous variables or the χ^2^ or Fisher test for categorical variables. SBP, systolic pressure; DBP, diastolic pressure; HbA1c, glycated Hemoglobin; FT3, free triiodothyronine; FT4, free thyroxine; TSH, thyroid-stimulating hormone; K^+^, serum potassium; Na^+^, serum sodium; Total Ca^2+^, serum total calcium; Cl^-^, serum chloride; Mg^2+^, serum magnesium; P, serum phosphorus.

Multivariate Cox regression analysis identified delayed medical care (7–30 days) (HR = 1.62, 95% CI: 1.02–2.56, *p* = 0.04), and a higher Wagner grade (HR = 1.62, 95% CI: 1.19–2.22, *p* = 0.003) as independent predictors of impaired DFU healing (**Table 5**).

**Table 5:** Multivariable Cox regression analysis of factors influencing diabetic foot ulcer healing.

Variables	HR (95% CI)	*p-*value
Wanger grades		
1–2 grades 3–5 grades	1.62 (1.19-2.22)	0.003
Duration of DFUs (days)		
≤7 days (7-30) days	1.62 (1.02-2.56)	0.04
Age (years)	1.01 (0.99-1.04)	0.41
Gender	1.00 (0.78-1.28)	0.98
Duration of diabetes (years)	1.01 (0.96-1.06)	0.65
Serum Albumin (g/L)	1.03 (0.98-1.07)	0.14

## Discussion

### Study summary

Most patients with DFUs resort to folk remedies or delay appropriate medical intervention, reflecting a critical gap between existing health education initiatives and patients’ health literacy. The delayed medical care (7–30 days) is associated with an increased risk of non-healing in DFUs. Prolonged pre-hospital delays were significantly associated with adverse clinical outcomes in patients with DFUs (6), supporting a biological gradient in which every additional week of pre-admission delay and every incremental Wagner stage translate into a quantifiable loss of regenerative opportunity. Kaplan-Meier survival analysis revealed that the cumulative non-healing rate curve for the DMCG persistently lay above that of the TMCG. Therefore, health education emphasizing timely medical consultation is of paramount importance for patients with DFUs.

### Barriers to timely medical intervention

The most common self-treatment approaches prior to hospitalization included no treatment, the application of Chinese herbal medicine, and the use of topical Western medications. Currently, there is a lack of high-quality evidence supporting the efficacy of topical Chinese herbal medicine for DFU management, with the majority of outcomes rated as low or moderate quality (10). Furthermore, only a small proportion of patients sought simple treatments at non-specialized centers. It is well established that tertiary hospitals possess extensive expertise and advanced technologies for treating diabetic foot diseases. However, among the 116 patients in our study, only 20 initially presented to a specialized diabetic foot center. This delay may be associated with a poor prognosis.

### Clinical characteristics and metabolic risk

In our study, the DMCG exhibited lower hemoglobin levels and a significantly greater prevalence of hypoalbuminemia than the TMCG. Vlad et al. noted that hypoalbuminemia is a well-established risk factor for amputation in DFUs (11). Patients with DFUs who underwent amputation exhibited lower hemoglobin and serum albumin levels (12). Adequate serum albumin levels are vital for maintaining plasma oncotic pressure and mitigating tissue edema, thereby creating an optimal microenvironment for tissue repair and regeneration. Compared with the TMCG, the DMCG exhibited higher Wagner grades and amputation rates. Higher Wagner grades may reflect greater disease severity (4).

### The significance of multidisciplinary management

Healthcare institutions at all levels must adhere to recommended guidelines and provide comprehensive diabetes education. This empowers patients to recognize the dangers of DFUs and seek specialized care early at diabetic foot centers. This proactive approach is essential for optimizing outcomes and reducing the burden of DFU (11). At the diabetic foot center, patients with DFUs received comprehensive long-term treatment through a multidisciplinary approach. This involved the multidisciplinary collaboration among endocrinology, podiatric surgery, and nutrition departments, ensuring that all aspects of the patient’s condition were addressed. The endocrinology team focused on closely monitoring and controlling blood glucose levels. Podiatric surgeons managed wound care, including regular debridement to remove necrotic tissue, promote granulation, and, when necessary, perform more complex procedures such as amputation. They also employed advanced wound management techniques, such as negative pressure wound therapy, which facilitated exudate drainage and supported the growth of healthy tissue. Nutritionists played a vital role by providing dietary guidance to optimize the patient’s overall health in the healing process. In addition to the initial treatment, patients underwent continuous follow-up and consultation. They regularly visited the center periodically for wound dressing changes and recorded the healing of ulcers.

### Mechanisms of impaired healing

DFUs are often the culmination of long-standing metabolic dysregulation associated with diabetes (3, 13, 14). In this study, all patients had poorly controlled chronic conditions (diabetes, hyperlipidemia, hypertension) that increased DFU risk. At the time of admission, key metabolic and clinical indicators were suboptimal, and these abnormalities posed significant barriers to ulcer healing (3, 14). Specifically, the non-healing group exhibited higher HbA1c levels, as well as lower hemoglobin and serum albumin levels. Glucose metabolic disorders lead to negative nitrogen balance and excessive protein loss from chronic, non-healing wounds. In DFU management, optimizing nutritional status is crucial for creating a favorable healing environment. Without balanced nutrition, ulcers are unlikely to heal, and the time required for healing will be significantly prolonged (15).

### Limitations

Despite confirming pre-hospital delay greater than 7 days as an independent adverse prognostic factor for DFU healing, this study has notable inherent limitations. First, single-center retrospective enrolment leads to selection bias owing to exclusion of mild outpatient DFU patients, restricting population generalizability; second, unavailable detailed socioeconomic and psychological confounders cannot be adjusted in multivariate models; third, pre-hospital irregular medication distribution was only descriptively counted without quantitative correlation analysis with healing endpoints. Future large-scale, multicenter prospective investigations are required to replicate our findings and further explore unresolved research questions. Our current outcomes can only serve as preliminary observational evidence rather than definitive clinical conclusion.

## Conclusions

In conclusion, this retrospective cohort preliminarily indicates that pre-hospital delay exceeding 7 days and elevated Wagner grade correlate with aggravated DFU severity and suboptimal healing outcomes in hospitalized patients. Targeted diabetes education to facilitate early specialist visits within one week after foot ulcer emergence is proposed as a potential protective strategy. Given the single-center, retrospective limitations above, all conclusions need further external validation via multicenter prospective research before widespread clinical promotion.

## Data Availability

The raw data supporting the conclusions of this article will be made available by the authors, without undue reservation.
